# 72 Daily Physical Activity Moderates the Relationship Between Symptoms and Social Participation Outcomes After Burn Injury

**DOI:** 10.1093/jbcr/irae036.064

**Published:** 2024-04-17

**Authors:** Huan Deng, Cailin Abouzeid, Lauren Shepler, Mary D Slavin, Juan Herrera-Escobar, Lewis E Kazis, Colleen M Ryan, Jeffrey C Schneider

**Affiliations:** PM&R, Spaulding Rehabilitation Hospital, Harvard Medical School, Charlestown, Massachusetts; Spaulding Rehabilitation Hospital, Harvard Medical School, Boston, Massachusetts; Department of Health Law, Policy and Management, Boston University School of Public Health, Boston, MA; Brigham and Women’s Hospital, Harvard Medical School, Boston, Massachusetts; Massachusetts General Hospital/Shriners Children's, Boston, MA; PM&R, Spaulding Rehabilitation Hospital, Harvard Medical School, Charlestown, Massachusetts; Spaulding Rehabilitation Hospital, Harvard Medical School, Boston, Massachusetts; Department of Health Law, Policy and Management, Boston University School of Public Health, Boston, MA; Brigham and Women’s Hospital, Harvard Medical School, Boston, Massachusetts; Massachusetts General Hospital/Shriners Children's, Boston, MA; PM&R, Spaulding Rehabilitation Hospital, Harvard Medical School, Charlestown, Massachusetts; Spaulding Rehabilitation Hospital, Harvard Medical School, Boston, Massachusetts; Department of Health Law, Policy and Management, Boston University School of Public Health, Boston, MA; Brigham and Women’s Hospital, Harvard Medical School, Boston, Massachusetts; Massachusetts General Hospital/Shriners Children's, Boston, MA; PM&R, Spaulding Rehabilitation Hospital, Harvard Medical School, Charlestown, Massachusetts; Spaulding Rehabilitation Hospital, Harvard Medical School, Boston, Massachusetts; Department of Health Law, Policy and Management, Boston University School of Public Health, Boston, MA; Brigham and Women’s Hospital, Harvard Medical School, Boston, Massachusetts; Massachusetts General Hospital/Shriners Children's, Boston, MA; PM&R, Spaulding Rehabilitation Hospital, Harvard Medical School, Charlestown, Massachusetts; Spaulding Rehabilitation Hospital, Harvard Medical School, Boston, Massachusetts; Department of Health Law, Policy and Management, Boston University School of Public Health, Boston, MA; Brigham and Women’s Hospital, Harvard Medical School, Boston, Massachusetts; Massachusetts General Hospital/Shriners Children's, Boston, MA; PM&R, Spaulding Rehabilitation Hospital, Harvard Medical School, Charlestown, Massachusetts; Spaulding Rehabilitation Hospital, Harvard Medical School, Boston, Massachusetts; Department of Health Law, Policy and Management, Boston University School of Public Health, Boston, MA; Brigham and Women’s Hospital, Harvard Medical School, Boston, Massachusetts; Massachusetts General Hospital/Shriners Children's, Boston, MA; PM&R, Spaulding Rehabilitation Hospital, Harvard Medical School, Charlestown, Massachusetts; Spaulding Rehabilitation Hospital, Harvard Medical School, Boston, Massachusetts; Department of Health Law, Policy and Management, Boston University School of Public Health, Boston, MA; Brigham and Women’s Hospital, Harvard Medical School, Boston, Massachusetts; Massachusetts General Hospital/Shriners Children's, Boston, MA; PM&R, Spaulding Rehabilitation Hospital, Harvard Medical School, Charlestown, Massachusetts; Spaulding Rehabilitation Hospital, Harvard Medical School, Boston, Massachusetts; Department of Health Law, Policy and Management, Boston University School of Public Health, Boston, MA; Brigham and Women’s Hospital, Harvard Medical School, Boston, Massachusetts; Massachusetts General Hospital/Shriners Children's, Boston, MA

## Abstract

**Introduction:**

Prior literature shows that early physical and psychological symptoms predict late social participation outcomes after burn injury. However, there is little information on the role of survivor’s daily behavior as it relates to physical and psychological symptoms and social participation outcomes. This study examines if daily behaviors moderate the association of pain, depression, and anxiety symptoms with social participation outcomes after burn injury.

**Methods:**

Adult burn survivors living in the community were enrolled in a 6-month study. Participants completed weekly surveys assessing physical symptom of pain (Patient-Reported Outcomes Measurement Information System (PROMIS) Pain Intensity and Pain Interference), psychological symptoms of depression (Patient Health Questionnaire (PHQ)) and anxiety (PROMIS Anxiety), as well as social participation outcomes (Life Impact Burn Recovery Evaluation (LIBRE) Profile Social Interactions and Social Activities). Additionally, a smartphone app and phone logs automatically recorded participants’ daily behaviors, including number of steps, travel miles, activity minutes, sleep hours, and number of phone call and message contacts. Multilevel models controlling for demographic and burn injury variables were separately conducted for each daily behavior to examine its moderation effect in the association between symptoms and social participation outcomes.

**Results:**

A total of 24 burn survivors were included in this study with an average of 25 years after injury and 43% total body surface area burned. Lower (worse) LIBRE Social Interactions and LIBRE Social Activities scores were significantly associated with higher (worse) PROMIS Pain Intensity, PROMIS Pain Interference, PHQ, and PROMIS Anxiety scores (p < 0.05). Additionally, within-person daily number of steps and activity minutes significantly moderated the association between PROMIS Anxiety and LIBRE Social Activities (p < 0.001).

**Conclusions:**

The study findings indicate that survivors’ social participation outcomes are related to pain, depression, and anxiety symptoms after burn injury, and are buffered by daily physical activity.

**Applicability of Research to Practice:**

This study may inform future interventions that target physical activity to improve outcomes and promote recovery after burn injury.

